# Bypass of the Major Alkylative DNA Lesion by Human DNA Polymerase η

**DOI:** 10.3390/molecules24213928

**Published:** 2019-10-31

**Authors:** Myong-Chul Koag, Hunmin Jung, Yi Kou, Seongmin Lee

**Affiliations:** The Division of Chemical Biology and Medicinal Chemistry, College of Pharmacy, The University of Texas at Austin, 2409 University Avenue, TX 78712, USA; mckoag@gmail.com (M.-C.K.); hunmin.jung@utexas.edu (H.J.); yik109@hotmail.com (Y.K.)

**Keywords:** N7-methylguanine, DNA damage, translesion synthesis DNA polymerase, X-ray crystallography

## Abstract

A wide range of endogenous and exogenous alkylating agents attack DNA to generate various alkylation adducts. N7-methyl-2-deoxyguanosine (Fm7dG) is the most abundant alkylative DNA lesion. If not repaired, Fm7dG can undergo spontaneous depurination, imidazole ring-opening, or bypass by translesion synthesis DNA polymerases. Human DNA polymerase η (polη) efficiently catalyzes across Fm7dG in vitro, but its structural basis is unknown. Herein, we report a crystal structure of polη in complex with templating Fm7dG and an incoming nonhydrolyzable dCTP analog, where a 2′-fluorine-mediated transition destabilization approach was used to prevent the spontaneous depurination of Fm7dG. The structure showed that polη readily accommodated the Fm7dG:dCTP base pair with little conformational change of protein and DNA. In the catalytic site, Fm7dG and dCTP formed three hydrogen bonds with a Watson–Crick geometry, indicating that the major keto tautomer of Fm7dG is involved in base pairing. The polη-Fm7dG:dCTP structure was essentially identical to the corresponding undamaged structure, which explained the efficient bypass of the major methylated lesion. Overall, the first structure of translesion synthesis DNA polymerase bypassing Fm7dG suggests that in the catalytic site of Y-family DNA polymerases, small N7-alkylguanine adducts may be well tolerated and form the canonical Watson–Crick base pair with dCTP through their keto tautomers.

## 1. Introduction

Enzymatic DNA methylation (e.g., 5-methylation of cytosine by DNA methyltransferase [1] and 6-methylation of adenosine by m6A methyltransferase [2]) plays important roles in nucleotide metabolisms. Nonenzymatic methylation by exogenous and endogenous methylating agents (e.g., (S)-adenosylmethionine and N-methyl-N-nitrosourea) [3,4], however, gives rise to a wide variety of genotoxic DNA lesions, including O6-methylguanine and N7-methylguanine [5,6]. O6-methylguanine represents a minor yet highly mutagenic lesion due to its ability to disrupt Watson–Crick base pairing during replication [7,8,9,10]. N7-methyl-2-deoxyguanosine (m7dG) is the predominant alkylative DNA lesion found in cells [11,12]. The level of m7dG is several thousand adducts per human cell and increases with aging and smoking [12,13]. The formal positive charge at the N7-G position greatly weakens the C–N glycosidic bond, promoting spontaneous depurination to produce highly mutagenic abasic sites, which induce G-to-T transversion mutations [14,15,16]. In addition, the imidazole moiety of m7dG is susceptible to basic conditions and can undergo ring-opening, generating mutagenic methyl-formamidopyrimidine adducts [17,18,19]. In the nucleosome, C8 of m7dG can be attacked by nucleophilic moieties of histone to yield reversible protein–DNA cross-links [20]. The intact m7dG can be removed by alkylative DNA glycosylases such as AlkA in *Escherichia coli* and alkyladenine DNA glycosylase in humans [19,20,21]. These alkylative DNA glycosylases recognize and catalyze hydrolysis of m7dG to engender abasic sites, which are further processed by downstream base excision repair enzymes [15,21,22]. If not removed by the base excision repair pathway, the intact m7dG could be bypassed by DNA polymerases, especially translesion synthesis (TLS) DNA polymerases.

**Figure 1:**
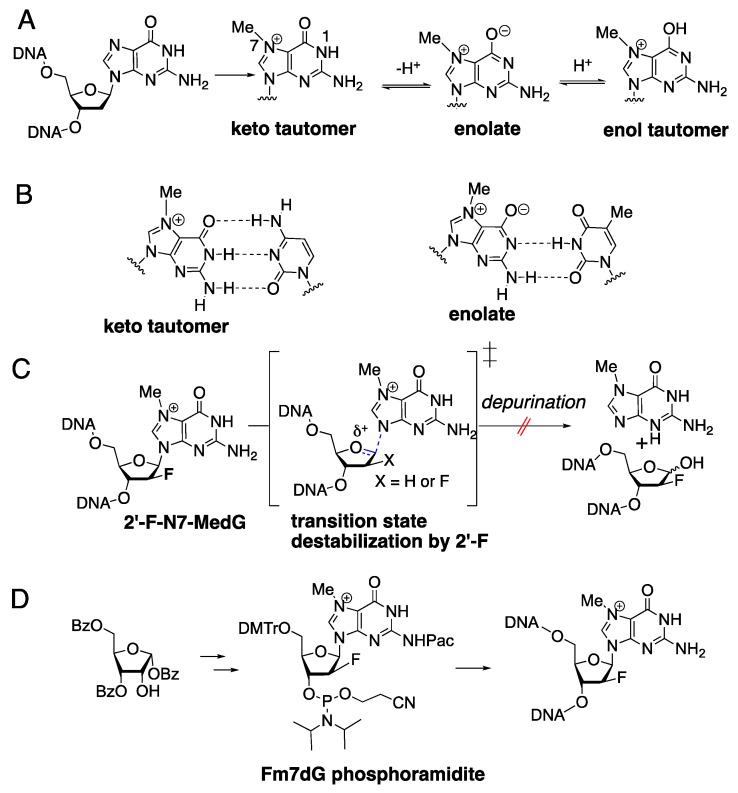
Base-pairing properties of N7-methyl-2-deoxyguanosine (m7dG). (**A**) Methylation of guanine and tautomers of m7dG. (**B**) Base pairings of the keto tautomer and the enolate of m7dG with dC and dT, respectively. (**C**) Prevention of the cleavage of the C–N glycosidic bond of m7dG via transition state destabilization. (**D**) Synthesis of N7-methyl-2′-fluorine-2′-deoxyguanosine (Fm7dG)-containing DNA from a ribose derivative. The 2′-fluorination prevented spontaneous depurination of Fm7dG and allowed site-specific incorporation of the lesion into DNA through solid-phase DNA synthesis and ultramild deprotection (50 mM K_2_CO_3_ in methanol at 25 °C).

While the mutagenic properties of secondary lesions (e.g., abasic sites and ring-opened products) of m7dG are well known, those of the primary m7dG lesion have been much less investigated. The presence of a positive charge at the guanine N7 position decreases pKa of N1-G by about two units [23,24]. This increases the population of the N1 ionized species at physiological pH, and the zwitterionic form of m7dG could favorably pair with thymine during DNA replication [25] (Figure 1A,B), which would promote G-to-A transition mutations. Systematic investigation of m7dG has been difficult due to the chemical instability of the m7dG nucleoside. A transition destabilization approach involving N7-methyl-2′-fluorine-2′-deoxyguanosine (Fm7dG; Figure 1C), a stable nonhydrolyzable m7dG analog, has enabled the site-specific incorporation of Fm7dG and the characterization of base-pairing properties of Fm7dG in duplex DNA [26,27,28,29,30]. In the catalytic site of polβ, Fm7dG and dCTP adopt a Watson–Crick base pair with three hydrogen bonds [28], indicating the keto tautomer of Fm7dG participates in correct base pairing. Despite the formation of the canonical Watson–Crick base pair conformation, dCTP incorporation opposite Fm7dG was found to be ~300-fold less efficient than that opposite dG, suggesting that m7dG may be a significant barrier to replication by some DNA polymerases that undergo an open-to-closed conformational reorganization. In the absence of protein contact, Fm7dG and dT form three hydrogen bonds with Watson–Crick-like geometry [27], indicating that the rare tautomer of m7dG engages in mutagenic m7dG:dT base pairing. Njuma et al. recently reported that Fm7dG is efficiently bypassed by TLS DNA polymerases such as human DNA polymerase η (polη) and *Solfolobus solfataricus* Dpo4 [29]. The catalysis across Fm7dG by TLS polymerases is error prone, producing 5%–10% of misincorporation products and decreasing the replication fidelity by ~5-fold.

Polη belongs to the Y-family DNA polymerases [31] and is well known for its efficiency to bypass various DNA lesions. Mutations in the *POLH* gene are found in xeroderma pigmentosum type V (XP-V) [32,33,34], an autosomal recessive disorder. XP-V patients are hypersensitive to UV radiation and have a higher mutation rate. Polη is specialized for the error-free bypass of UV-induced cyclobutene pyrimidine dimers through an enlarged active site [32]. Like other Y-family DNA polymerases, polη does not undergo an open-to-closed conformational change during translesion synthesis. Polη stabilizes damaged DNA in a normal B-DNA conformation through its “molecular-splint”-like action [35]. Polη reduces cellular sensitivity to a wide spectrum of DNA-damaging drugs such as platinum-based anticancer agents [36]. The enzyme has been also implicated in bypassing O6-methylguanine and 8-oxoguanine [9,37]. Herein, we report the kinetic results of polη incorporating dCTP or dTTP opposite templating Fm7dG that was site-specifically introduced by the transition destabilization approach. We also present a ternary complex structure of polη in complex with a nonhydrolyzable dCTP analog and templating Fm7dG. The first structure of TLS DNA polymerase bypassing the major methylated DNA lesions provides new insights into the promutagenicity of small N7-alkylguanine adducts.

## 2. Results and Discussion

### 2.1. Steady-State Kinetic Studies

**Figure 2:**
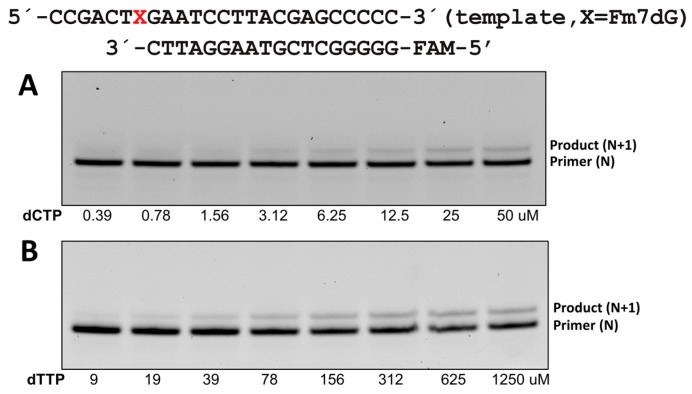
Incorporation of an individual nucleotide opposite templating Fm7dG by polη. A PAGE-urea gel for insertion of dCTP (**A**) or dTTP (**B**) opposite Fm7dG by polη. Polη, Fm7dG-containing 25 mer template, 18 mer primer, and incoming nucleotides were used for the reaction.

The steady-state kinetic studies of polη incorporating dCTP or dTTP opposite templating Fm7dG provided important insights into the impact of guanine N7-alkylation on promutagenic replication. For this study, we used the catalytic domain (amino acids 1–432) of polη, a 25 mer template DNA and an 18 mer primer DNA containing a 5′-fluorescein amidite (FAM) label (Figure 2). Polη incorporated dCTP opposite Fm7dG ~3-fold less efficiently than opposite dG (45.6 vs. 13.2 10^−3^ s^−1^ μM^−1^), showing that the presence of the formal positive charge at N7-G and the methyl moiety did not dramatically reduce the catalytic efficiency of polη (Table 1). *K*_m_ for the Fm7dG:dCTP insertion slightly increased compared with that for the dG:dCTP insertion, whereas *k*_cat_ decreased about 2-fold (120.6 vs. 56.4 10^−3^ s^−1^). Misincorporation of dTTP opposite templating Fm7dG was ~14-fold (0.94 vs. 13.2 10^−3^ s^−1^ μM^−1^) less efficient than dCTP insertion opposite the lesion. The decrease in the catalytic efficiency for Fm7dG:dTTP insertion was mainly caused by the increase in *K*_m_ (4.27 vs. 52.5 μM). Interestingly, polη incorporated dTTP opposite Fm7dG ~2-fold (0.94 vs. 0.47 10^−3^ s^−1^ μM^−1^) more efficiently than opposite dG, suggesting that Fm7dG promotes mutagenic base pairing. Importantly, the replication fidelity for Fm7dG decreased by ~7-fold (14.3 vs. 97.0) compared with that for dG, suggesting that major methylated DNA lesions significantly promote error-prone replication.

### 2.2. Structure of Polη Incorporating dCTP Opposite Templating Fm7dG

**Figure 3:**
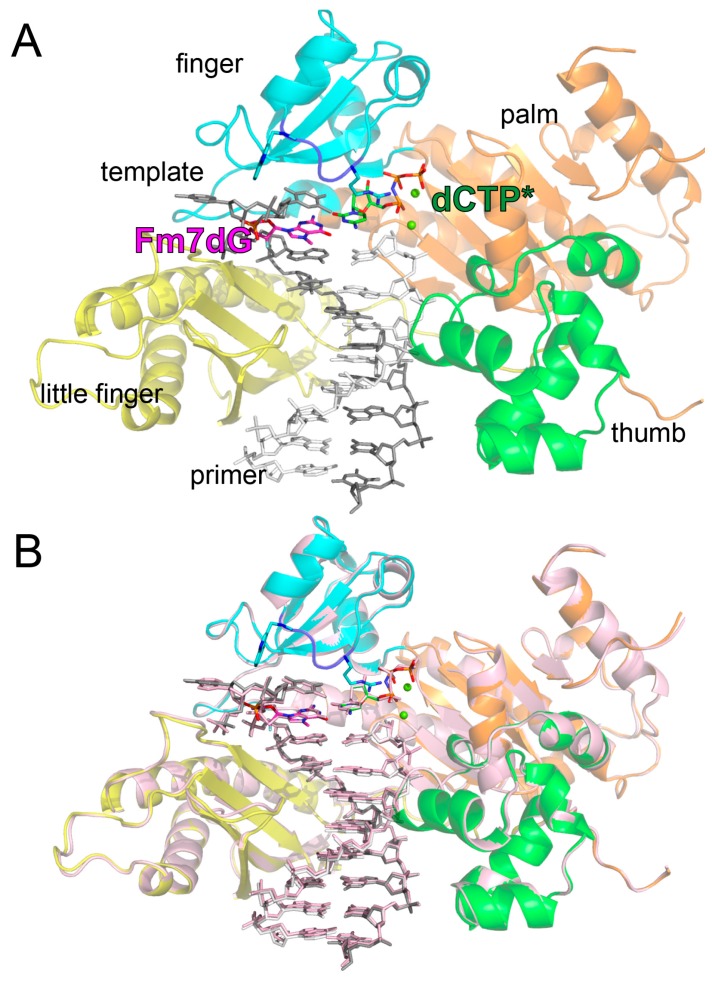
Overall conformation of the polη-Fm7dG:dCTP* complex structure. (**A**) The ternary complex structure of polη bound to Fm7dG and the incoming nonhydrolyzable dCTP analog. The palm, thumb, finger, and little finger domains are shown in orange, green, cyan, and yellow, respectively. The flexible Arg61–Trp64 loop is shown in blue. The primer strand is colored in white and the template strand in gray. The active site Mg^2+^ ions are shown in green spheres. (**Β**) Superposition of the polη-Fm7dG:dCTP* and the polη-dG:dCTP* (light pink, PDB ID: 4O3N [41]) structures.

To gain structural insights into how TLS DNA polymerases bypass the major alkylative DNA lesion, we determined a ternary structure of polη in complex with templating Fm7dG paired with incoming nonhydrolyzable dCMPNPP (hereinafter dCTP*) (Figure 3A). The nonhydrolyzable dCTP analog was used for this crystallographic study because it is isosteric to the natural nucleotide and its coordination with Mg^2+^ ions is virtually identical to that of dCTP. The NH moiety of dCMPNPP replaces the bridging oxygen between P_α_ and P_β_, making the analog resistant to dCMP transfer as well as hydrolysis, which enables the capture of a ternary complex of a catalytically active polη bound to Fm7dG:dCTP* in the presence of Mg^2+^. This also allows the coordination of the primer terminus 3′-OH to the catalytic metal ion. The nonhydrolyzable dNTP* analog has been used in crystallographic studies of various DNA polymerases [38,39,40]. The polη-Fm7dG:dCTP* ternary complex was crystallized in the P61 space group with the cell dimension of a = b = 98.7 Å, c = 81.8 Å, α = β = 90.0°, and γ = 120° and one protein in the asymmetric unit. The polη ternary complex structure was refined to 2.3 Å with R_work_ = 17.1% and R_free_ = 23.3%.

The overall conformation of the polη-Fm7dG:dCTP* ternary structure is essentially identical to that of the published ternary structure with dG:dCTP insertion (PDB ID: 4O3N) [41]: The root-mean-square deviation for these structures was 0.211 Å. (Figure 3B). The polη-Fm7dG:dCTP* structure displayed the thumb, palm, finger, and little finger domains of Y-family DNA polymerases (Figure 3A). The incoming nucleotide resided between the palm and finger domains, whereas the templating Fm7dG was positioned between the finger and little finger domains. The statistics for data collection and the refinement are summarized in Table 2.

**Figure 4:**
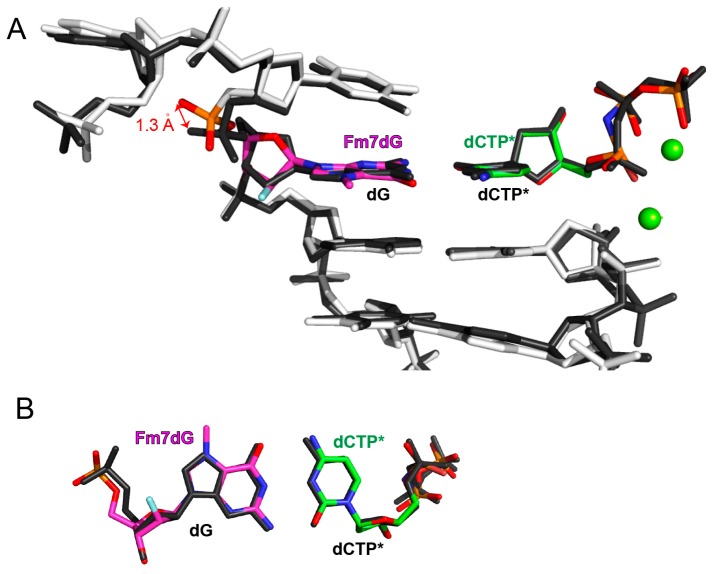
Superpositions of DNA conformation of the polη-Fm7dG:dCTP* and published polη-dG:dCTP* structures. (**A**) Superposition of the template and primer strands of the polη-Fm7dG:dCTP* and polη-dG:dCTP* structures. The distance between the nonbridging oxygens of phosphodiester is indicated. (**B**) Superposition of the replicating base pairs of the polη-Fm7dG:dCTP* and polη-dG:dCTP* structures.

The conformational difference between the polη-Fm7dG:dCTP* and published polη-dG:dCTP* structures (PDB ID: 4O3N) was confined to Fm7dG and the upstream of Fm7dG (Figure 4A); their protein conformations were essentially the same (Figure 3B). The nonbridging phosphate oxygen of the 5′-phosphodiester of Fm7dG shifted 1.3 Å relative to the dG:dCTP* structure (Figure 4B). Slight conformational difference was also observed at the 5′ side of the templating bases. It appears that the spacious catalytic site of polη well accommodates small alkyl-dG lesions with little distortion in protein and DNA conformations.

**Figure 5:**
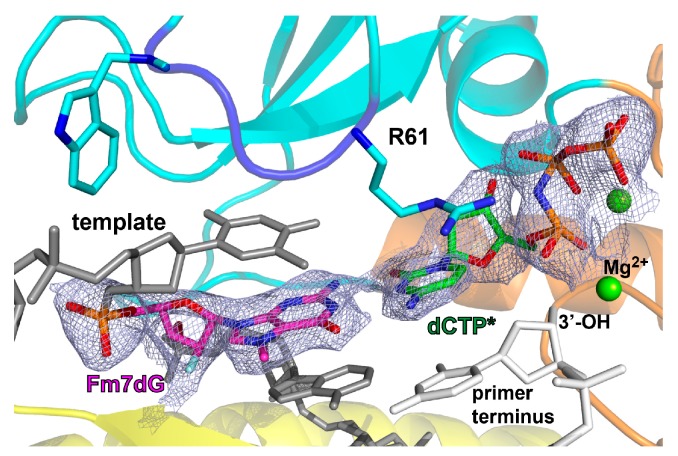
Polη accommodating the Fm7dG:dCTP* base pair in the catalytic site. Close-up view of Fm7dG:dCTP* in the replicating base pair site of polη. A *2Fo-Fc* electron density map contoured at 1σ around Fm7dG:dCTP* is shown. The active site Mg^2+^ ions are shown in green spheres. Arg61, which interacts with the incipient base pair, is shown in cyan sticks. The color scheme is the same as in Figure 3A. The Arg61–Trp64 loop is shown in blue.

The ternary complex structure revealed the base-pairing characteristics of Fm7dG and dCTP* in the replicating base pair site (Figure 5). A 2*F_o_* − *F_c_* electron density map contoured at 1σ around Fm7dG:dCTP* clearly showed the presence of the N7 methyl moiety and 2′-β-fluorine of Fm7dG and incoming dCTP*. Polη tolerated the Fm7dG:dCTP* base pair with little distortion of the catalytic site. In particular, the flexible Arg61–Trp64 loop [32,36,38,42,43], which undergoes a significant conformational change upon binding of bulky DNA lesions (e.g., cyclobutene pyrimidine dimers, cisplatin-GpG, oxaliplatin-GpG, and phenanthriplatin-G), adopted essentially the same conformation as observed in the undamaged structure. In the nascent base pair site, templating Fm7dG formed a coplanar base pair with dCTP* and engaged in stacking interactions with the adjacent 5′ and 3′ bases (Figure 5). The guanidinium moiety of Arg61 was oriented toward and stacked with the incoming dCTP*.

**Figure 6:**
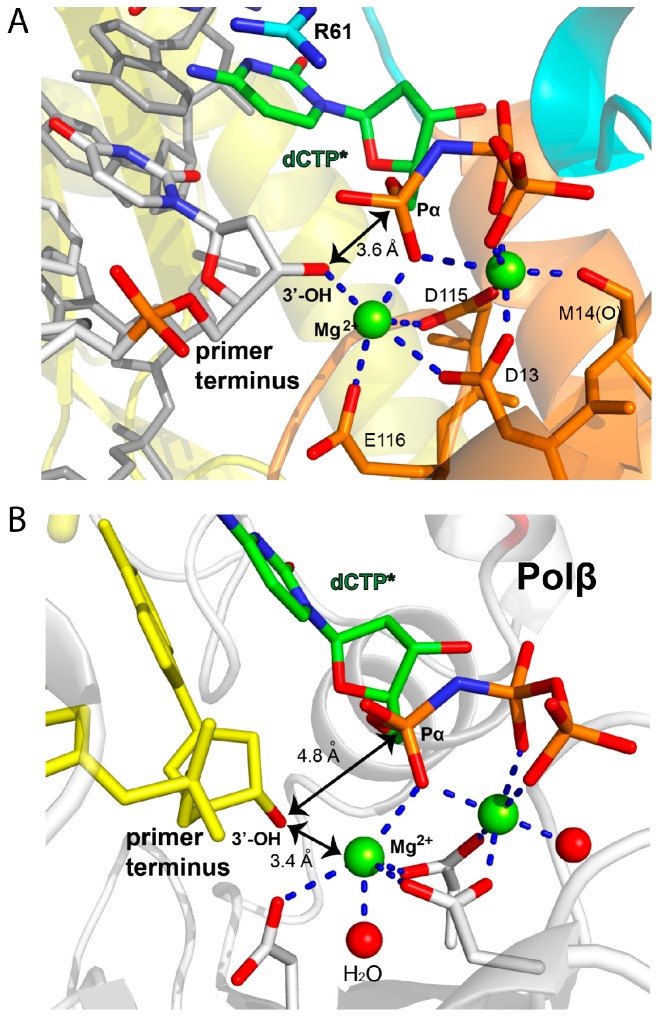
Metal coordination of polymerase-Fm7dG:dCTP* ternary structures. (**A**) Coordination of the catalytic and nucleotide-binding metal ions in the polη-Fm7dG:dCTP* structure. The distance between the 3′-OH of the primer terminus and the P_α_ of dCTP* is indicated as a double-headed arrow. The coordination of metal ions is indicated as dashed lines. Note that the 3′-OH is optimally positioned for nucleotidyl transfer. (**B**) Metal coordination in the published polβ-Fm7dG:dCTP* ternary complex structure (PDB CODE: 4O5K). The primer terminus 3′-OH is not coordinated to the catalytic metal ion, 4.8 Å away from the P_α_ of dCTP*, and poorly positioned for in-line nucleophilic attack.

A close-up view of the metal-binding site gives insights into the facile bypass of Fm7dG by polη (Figure 6A, Table 1). Both the catalytic “metal A” and nucleotide-binding “metal B” ions were observed in the active site. The catalytic Mg^2+^ ion was coordinated with the 3′-OH of the primer terminus, a nonbridging oxygen of P_α_, and catalytic carboxylates (Asp13, Asp115, and Glu116). The nucleotide-binding metal ion was complexed with Asp13, Met14, Asp115, and nonbridging oxygens of P_β_ and P_γ_. The nucleophilic 3′-hydroxyl group of the primer terminus was 3.6 Å from the P_α_ and poised for the in-line nucleophilic attack on the P_α_, which would facilitate nucleotidyl transfer. Overall, the observation of the Fm7dG:dCTP* base pair with an ideal Watson–Crick geometry, together with the favorable metal ion coordination for catalysis, is consistent with the efficient incorporation of dCTP opposite Fm7dG by the enzyme (Table 1) [29].

The methyl modification on guanine N7 decreased the catalytic efficiency of polη and polβ by ~3- and ~300-fold, respectively, which suggests a differential impact of Fm7dG on polymerase activity [28]. The dramatic difference in the catalytic efficiency may have resulted from the variation in the sensitivity of polymerases toward the lesion. In the case of polβ, while Fm7dG:dCTP* formed the canonical Watson–Crick base pairing (Figure 6B), coordination [44,45] between the 3′-OH of the primer terminus and the catalytic metal ion was lacking. In addition, the distance between the primer terminus 3′-OH and the P_α_ of dCTP* was longer than that for correct insertion (4.8 Å vs. ~3.4 Å). Furthermore, the nucleophilic 3′-OH was pointed away from the P_α_ of dCTP*, thereby assuming a catalytically unfavorable conformation. This nonoptimal conformation for nucleotidyl transfer would slow the catalysis by polβ [28]. The polβ-Fm7dG:dCTP* complex would require conformational adjustments to reach the catalytically competent state, which may be a slow or rate-limiting step in catalysis. In the case of polη, the metal ion coordination for the polη-Fm7dG:dCTP* complex was essentially identical to that for the polη-dG:dCTP* complex and optimally positioned for catalysis.

**Figure 7:**
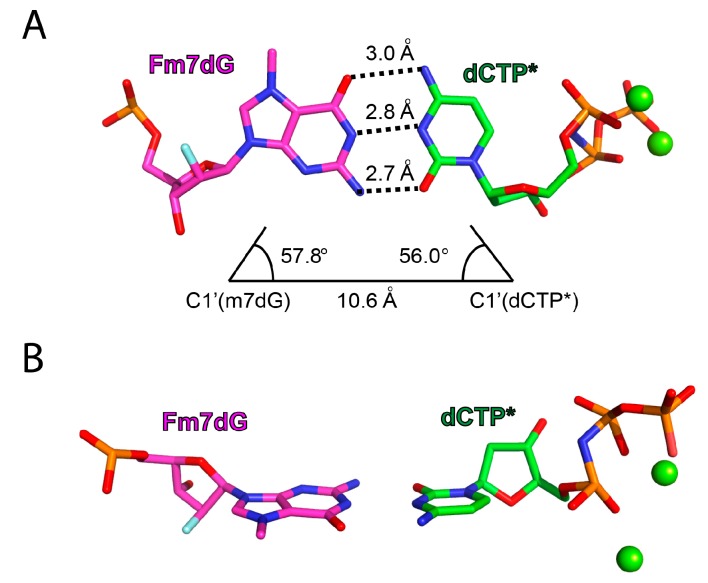
Base-pairing properties of Fm7dG and dCTP* in the replicating base pair site of polη. (**A**) Watson–Crick base pairing of Fm7dG:dCTP*. The interbase hydrogen bonds are indicated with dotted lines. The λ angles and C1′–C1′ distance for Fm7dG:dCTP* are shown. (**B**) Side view of Fm7dG:dCTP* with a coplanar base pair conformation.

The base-pairing characteristics of Fm7dG:dCTP* were very similar to those of dG:dCTP* (Figure 7). In the polη active site, Fm7dG:dCTP* adopted the canonical Watson–Crick base pair with an average hydrogen bond distance of 2.8 Å (Figure 7A). Specifically, the O6, N1, and N2 of Fm7dG were hydrogen bonded to the N4, N3, and O2 of dCTP*, respectively, with distances of 3.0, 2.8, and 2.7 Å. The C1′–C1′ distance for Fm7dG:dCTP* was 10.6 Å and the λ angles for Fm7dG and dCTP* were 57.8° and 56.0°, respectively, which were almost the same as observed in normal Watson–Crick base pairing. The b-factor value of the nucleobases of Fm7dG and dCTP* was in the 9–15 range (not shown), illustrating that the Fm7dG:dCTP* base pair was well ordered in the nascent base pair site. The propeller-twist angle for Fm7dG:dCTP* was ~10°, signifying that Fm7dG did not significantly distort base pair conformation (Figure 7B). Altogether, the Fm7dG:dCTP* base pair was virtually indistinguishable from the dG:dCTP base pair, indicating that the positively charged N7 methyl moiety negligibly alters the base-paring property during correct nucleotide incorporation.

The formation of Fm7dG:dCTP* with Watson–Crick geometry strongly suggests that, in the polη active site, the keto tautomer of Fm7dG, rather than the zwitterionic or enol tautomeric form of Fm7dG, exists as the major tautomer (Figure 1A). The enolate or enol tautomer of Fm7dG has been observed when a lesion is paired with dT in the absence of polymerase contact [27]. The suppression of zwitterionic species could arise from the electron-rich microenvironment (e.g., bases and phosphate anions) around Fm7dG, which can diminish the impact of the positively charged N7-methyl group on the ionization of N1 of Fm7dG.

In summary, the first structure of TLS polymerase catalyzing across a major methylated DNA lesion suggests that small N7-alkylguanine adducts can be readily accommodated in the catalytic site of TLS polymerases. In the polη active site, Fm7dG and dCTP* form three hydrogen bonds with an ideal Watson–Crick geometry, which explains the highly efficient nucleotidyl transfer opposite Fm7dG. It is highly likely that small N7-alkylguanine lesions engage in the canonical Watson–Crick base pairing with dCTP through their keto tautomers. It would be of interest to evaluate the impact of the steric bulkiness of the alkyl moiety (e.g., ethyl, benzyl, and nitrogen half mustard) on the base pairing characteristics of N7-alkylguanine lesions.

## 3. Materials and Methods

### 3.1. Synthesis of Fm7dG-Containing Oligonucleotide

The Fm7dG phosphoramidite was synthesized starting from a ribose derivative according to the synthetic procedures described previously [26]. The site-specific incorporated Fm7dG-containing DNA was custom synthesized by Midland Certified Reagent company (Midland, TX).

### 3.2. Cloning and Protein Expression and Purification

The catalytic core of human polη (amino acids 1–432) was cloned into pET28a plasmid with NcoI and BamHI restriction enzyme sites. *E. coli* BL21 (DE3) cells transformed with this plasmid were grown at 37 °C in LB medium supplemented with 50 μg/mL until the OD600 of 0.6. Protein expression was induced for 18 h at 20 °C by adding 0.3 mM isopropyl-β-thiogalactoside. Cells were collected by centrifugation at 8000× *g* for 20 min at 4 °C. Proteins were purified by Ni^2+^-NTA affinity, Heparin column, and Superdex-75 gel filtration chromatography. Purified human polη was concentrated to 15 mg/mL in a gel filtration buffer (25 mM Tris, pH 7.5, 300 mM KCl, 10% glycerol, and 2 mM dithiothreitol), aliquoted, and flash-frozen in liquid nitrogen to store at −80 °C.

### 3.3. Steady-State Kinetics of Single Nucleotide Incorporation Opposite Templating Fm7dG by Polη

Steady-state kinetic parameters for insertion opposite Fm7dG by polη were measured as described previously, with slight modifications [42]. The oligonucleotides for kinetic assays (primer, 5’-FAM/GGGGGCTCGTAAGGATTC-3′ and template, 5´-CCGACT(Fm7dG)GAATCCTTACGAGC CCCC-3´) were synthesized by Midland Certified Reagent company (Midland, TX, USA) and Integrated DNA Technologies (Coralville, IA, USA). To prepare the duplex DNA substrate for polη, both oligonucleotides were annealed in hybridization buffer (10 mM Tris-HCl, pH 7.5; 1 mM EDTA) at 90 °C for 5 min. Enzyme activities were determined using the reaction mixture containing 40 mM Tris-HCl (pH 8.0), 60 mM KCl, 10 mM dithiothreitol (DTT), 250 μg/mL bovine serum albumin (BSA), 2.5% glycerol, 5 mM MgCl_2_, 50–100 nM recessed DNA (both control dG and Fm7dG), and varying concentrations of incoming dNTP. To prevent end-product inhibition and substrate depletion from interfering with accurate velocity measurement, the enzyme concentrations and reaction times were adjusted for each experiment (less than 20% insertion product formed). The reactions were initiated by the addition of the enzyme and stopped with a gel-loading buffer (95% formamide with 20 mM EDTA, 45 mM Tris-borate, 0.1% bromophenol blue, and 0.1% xylene cyanol). The quenched samples were separated on 20% denaturing polyacrylamide gels. The gels were visualized and analyzed using Typhoon Imager (GE Healthcare Life Sciences) to quantify product formation. The *k*_cat_ and *K*_m_ were determined by fitting the reaction rate over dNTP concentrations to the Michaelis–Menten equation. Each experiment was repeated three times to measure the average and the standard deviation of the kinetic results. The efficiency of nucleotide insertion was calculated as *k_cat_/K_m_*. The relative frequency of dNTP incorporation opposite Fm7dG was determined as *f* = (*k*_cat_/*K_m_*)_[dN:Fm7dG]_ /(*k*_cat_/*K_m_*)_[dC:dG]_.

### 3.4. Crystallization, Data Collection, and Refinement

The template oligonucleotides for X-ray crystallographic studies (5′-CAT(**X**)ATGACGCT-3′, X = dG or Fm7dG) were synthesized by Midland Certified Reagent Co. (Midland, TX, USA). The primer, 5′-AGCGTCAT-3′, was synthesized by Integrated DNA Technologies (Coralville, IA, USA). The oligonucleotides were mixed at a 1:1 molar ratio and annealed in HEN buffer (10 mM HEPES, pH 8.0; 0.1 mM EDTA; and 50 mM NaCl) by heating for 10 min at 90 °C and slowly cooling to room temperature. The cocrystals of the ternary complex of polη-Fm7dG:dCTP* were grown at conditions similar to those described previously [46]. Briefly, the crystal was obtained by using the hanging drop vapor diffusion method in a reservoir buffer containing 0.1 M MES (pH 5.5), 5 mM MgCl_2_, and 15%–20% PEG 2K-MME. Diffraction data were collected on 3–5-day-old crystals that were cryoprotected with 20% glycerol at 100 K using the beamline 5.0.3 at the Advanced Light Source, Lawrence Berkeley National Laboratory. All diffraction data were processed using HKL 2000 (HKL research, Charlottesville, VA, USA) [47]. Structures were solved by molecular replacement using the polη-dG:dCTP* structure (PDB code 4O3N) as the search model [41]. The model was built using COOT [48] and refined using Phenix software [49]. MolProbity was used to make Ramachandran plots [50].

## Figures and Tables

**Table 1:** Kinetic parameters for nucleotide incorporation opposite Fm7dG and dG by polymerase η (polη).

Template:dNTP	*K*_m_ (μM)	*k*_cat_ (10^−3^ s^−1^)	*k*_cat_/K_m_ (10^−3^ s^−1^μM^−1^)	*f* ^a^	Replication Fidelity
dG:dCTP	2.66 ± 0.29	120.6 ±6.1	45.6	1	
dG:dTTP	159.3 ± 2.7	74.8 ± 0.9	0.47	0.01	97
Fm7dG:dCTP	4.27 ± 0.42	56.4 ± 2.7	13.2	1	
Fm7dG:dTTP	52.5 ± 1.7	49.3 ± 0.05	0.94	0.07	14.3

^a^ Relative efficiency: (*k*_cat_/*K*_m_)_[dTTP:dG]_/(*k*_cat_/*K*_m_)_[dCTP:dG]_ or (*k*_cat_/*K*_m_)_[dTTP:Fm7dG]_/(*k*_cat_/*K*_m_)_[dCTP:Fm7dG]_.

**Table 2:** Data collection and refinement statement statistics.

**PDB CODE**	Fm7dG:dCTP* **(6UI2)**
Data Collection	
space group	*P*61
Cell Constants	
a (Å) b c α (°) β γ	98.684 98.684 81.852 90 90 120
resolution (Å)^a^	20–2.34 (2.39–2.34)
*R*_merge_^b^ (%)	0.113 (0.441)
<I/σ>	20.4 (5.28)
completeness (%)	100.0 (100.0)
redundancy	11.3 (11.3)
**Refinement**	
*R*_work_^c^/*R*_free_^d^ (%)	17.1/23.3
unique reflections	19,191
Mean B Factor (Å^2^)	
protein	24.94
ligand	23.51
solvent	26.49
Ramachandran Plot	
most favored (%)	96.2
add. allowed (%)	3.3
RMSD bond lengths (Å) bond angles (degree)	0.009 1.55

^a^ Values in parentheses are for the highest resolution shell; ^b^ *R*_merge_ = Σ|I − <I>|/ΣI, where I is the integrated intensity of a given reflection; ^c^ *R*_work_ = Σ|F(obs) − F(calc)|/ΣF(obs); ^d^*R*_free_ = Σ|F(obs) − F(calc)|/ΣF(obs), calculated using 5% of the data.
